# Effect of Cyclodextrins Formulated in Liposomes and Gold and Selenium Nanoparticles on siRNA Stability in Cell Culture Medium

**DOI:** 10.3390/ph17101344

**Published:** 2024-10-08

**Authors:** Betzaida Castillo Cruz, Sandra Chinapen Barletta, Bryan G. Ortiz Muñoz, Adriana S. Benitez-Reyes, Omar A. Amalbert Perez, Alexander C. Cardona Amador, Pablo E. Vivas-Mejia, Gabriel L. Barletta

**Affiliations:** 1 Department of Chemistry, University of Puerto Rico at Humacao, Humacao 00791, Puerto Rico; betzaida.castillo1@upr.edu (B.C.C.); bryan.ortiz20@upr.edu (B.G.O.M.); adriana.benitez@upr.edu (A.S.B.-R.); omar.amalbert@upr.edu (O.A.A.P.); alexander.cardona@upr.edu (A.C.C.A.); 2 Department of Physiology/Pathology, San Juan Bautista School of Medicine, Caguas 00725, Puerto Rico; schinapen@sanjuanbautista.edu; 3 Department of Biochemistry, University of Puerto Rico Medical Sciences Campus, San Juan 0035, Puerto Rico; 4 Comprehensive Cancer Center, University of Puerto Rico, San Juan 00936, Puerto Rico

**Keywords:** cyclodextrins, liposomes, gold nanoparticles, selenium nanoparticles, siRNA stabilization and delivery

## Abstract

Background: Encapsulation of siRNA fragments inside liposome vesicles has emerged as an effective method for delivering siRNAs in vitro and in vivo. However, the liposome’s fluid-phospholipid bilayer of liposomes allows siRNA fragments to diffuse out of the liposome, decreasing the dose concentration and therefore the effectiveness of the carrier. We have previously reported that β-cyclodextrins formulated in liposomes help increase the stability of siRNAs in cell culture medium. Here, we continued that study to include α, γ, methyl-β-cyclodextrins and β-cyclodextrin-modified gold and selenium nanoparticles. Methods: We used Isothermal Titration Calorimetry to study the binding thermodynamics of siRNAs to the cyclodextrin-modified nanoparticles and to screen for the best adamantane derivative to modify the siRNA fragments, and we used gel electrophoresis to study the stabilization effect of siRNA by cyclodextrins and the nanoparticles. Results: We found that only β- and methyl-β-cyclodextrins increased siRNA serum stability. Cyclodextrin-modified selenium nanoparticles also stabilize siRNA fragments in serum, and siRNAs chemically modified with an adamantane moiety (which forms inclusion complexes with the cyclodextrin-modified-nanoparticles) show a strong stabilization effect. Conclusions: β-cyclodextrins are good additives to stabilize siRNA in cell culture medium, and the thermodynamic data we generated of the interaction between cyclodextrins and adamantane analogs (widely used in drug delivery studies), should serve as a guide for future studies where cyclodextrins are sought for the delivery and solvation of small organic molecules.

## 1. Introduction

In vivo delivery of bioactive compounds remains a challenge in medicine, as many drugs suffer from poor aqueous solubility, the inability to permeate cell membranes, poor stability in serum and a lack of specificity toward the target tissue [1]. Small interfering RNA (siRNA) is a therapeutic modality for preventing the synthesis of proteins that are aberrantly abundant in many diseases, including cancer [2,3]. siRNAs use small oligonucleotide RNAs that bind mainly to the 3’ UTR of a messenger RNA (mRNA), in this way avoiding protein synthesis [2,3]. This novel modality holds significant promise for treating a range of diseases, particularly those driven by genetic mutations or aberrant gene expression. However, siRNA molecules are quickly degraded in serum by nucleases, cannot permeate the cell membrane, and cannot discriminate among tissues [4,5,6]. Nanocarriers have been used extensively during the last decade for transporting siRNAs to their targets. However, to be effective, nanocarriers must bind siRNA fragments, protect them from nucleases in the medium, target the tissue of interest, permeate the cell membrane and release the siRNA cargo inside the cell cytoplasm. This requires nanocarriers to be modified with several task-specific groups, resulting in complex synthetic and purification steps [7]. Recently, liposome formulations have gained much attention as promising siRNA delivery vehicles because of their ease of preparation and modification; each phospholipid that makes up a liposome can be individually modified with a particular task-specific group, representing an advantage over the synthesis of most nanoparticles, where each nanoparticle must be chemically modified with all necessary groups [8,9]. However, siRNAs can leach out liposomes immediately after they are prepared, drastically decreasing the dose concentration over time and hence the effectiveness of the carrier [10]. Generally, they are completely depleted from liposomes within 48 h or less upon incubation in cell culture medium [10]. We previously reported that β-cyclodextrins (βCDs) incorporated in a liposome formulation minimize the diffusion of siRNAs through a liposome’s membrane, increasing their potential as siRNA delivery vehicles [10]. This phenomenon can be explained in part by a theoretical study reported by R. Zappacosta et al. [11], who concluded that βCD has difficulty permeating a liposome’s membrane owing to H-bond interactions with surface phosphate groups of the phospholipids [11]. Furthermore, via isothermal titration calorimetry (ITC), we showed that βCDs do not form inclusion complexes with double-stranded siRNA fragments [10]. Taken together, these results suggest a mechanism by which βCD attaches to the surface of siRNA fragments, probably by H-bonding, and prevents it from permeating the liposome membrane [10]. The goal of the current work was to find other cyclodextrin (CD) molecules and nanoparticles modified with cyclodextrins to improve the stability of siRNA fragments in cell culture media. To this end, we studied the effects of α, γ and methyl-β-cyclodextrins (αCD, γCD and MeβCD, respectively), as well as gold (Au) and selenium (Se) β-cyclodextrin-modified nanoparticles (AuβCD, SeβCD), on the stability of siRNA incubated in 50% serum. We also studied the stability in serum of adamantane-siRNA-modified fragments (AD-siRNAs) formulated with AuβCD and SeβCD. Adamantane (AD) is composed of six cyclohexane rings fused together, forming a compact structure that measures 6.8 Å wide, 7.6 Å in length and 7.4 Å in height, with a volume of 159.43 Å^3^ [12]. AD is hydrophobic and fits inside the hydrophobic cavity of β-cyclodextrins, which have an opening of 7.8 Å and a volume of 262 Å^3^ [13]. As our data show, AD strongly binds to β-cyclodextrins; therefore, it can be used as an anchor for attaching large molecules to β-cyclodextrins to construct larger structures such as nanoparticles. In this study, we modified siRNA fragments with an adamantane derivative to bind β-cyclodextrins to the siRNAs or to bind the AD-modified siRNA fragments to nanoparticles made of gold or selenium modified with β-cyclodextrin on their surface. As previously reported, siRNA fragments are degraded upon incubation at 37 °C for 24 h in 50% serum [10]. When the fragments are encapsulated in a 1,2-dioleoyl-sn-glycero-3-phosphocholine (DOPC) liposome, their lifetime in serum is extended for over 24 h [10,14]. This stabilization effect is extended even further when β-cyclodextrins are added to the siRNA-liposome formulation [10]. Here, we report that methyl-β-cyclodextrins formulated into a siRNA-liposome offer a better stabilization effect than do β-cyclodextrins. However, γ-cyclodextrins showed a far lower stabilization effect, and surprisingly, α-cyclodextrins were detrimental to the liposome formulation, as most of the siRNA fragments were degraded after a 48-hour incubation period in 50% serum. SeβCD nanoparticles also substantially stabilized siRNAs, whereas AuβCDs were not very effective at stabilizing siRNA fragments. In addition to the number of glucopyranose units, these cyclodextrins have different propensities to form inter- and intra-H-bonds, which could shape how they interact with siRNA fragments and the phospholipid membrane of liposomes [13]. β-Cyclodextrin forms a secondary belt of H-bonds between the C2-OH and C3-OH of adjacent glucopyranose units, resulting in these cyclodextrins having a reduced ability to form H-bonds with surrounding water molecules, an effect demonstrated by their low water solubility; βCDs are the least water soluble than all the other natural cyclodextrins (1.8 mg/mL). Methyl-β-cyclodextrin, on the other hand, has around 63% of its C2-OH, C3-OH (and primary -OH) methylated (C-O-CH_3_), disrupting the H-bond belt. Note: This means that approximately 1.9 out of 3 -OH groups are methylated per mol of glucose, with a total molecular weight of 1320 g/mol. This cyclodextrin is the most water soluble of the group (around 50 mg/mL), and although its shape (truncated cone [15] or hyperboloid of revolution [16]) and cavity size are identical to those of β-cyclodextrin, its methoxy groups make the inner cavity highly hydrophobic [17]. Another important characteristic of methyl-β-cyclodextrin, and to some degree of β-cyclodextrin, that could be a factor in the siRNA-inside-a-liposome stabilization effect we observe [10] is that they bind cholesterol [18,19]. Cholesterol is found on cell membranes and is a critical ingredient in liposome formulations. Its depletion from the liposomal membrane by cyclodextrins could be a factor in the stabilization effect we observed. Studies addressing this topic are underway in our laboratory. Unlike all other cyclodextrins, γ-cyclodextrin has a distorted-truncated cone shape, which prevents H-bonding between the C2-OH and C3-OH of different glucopyranose units, hence disrupting the H-bond belt [13]. Also, α-cyclodextrins do not possess a belt of H-bonds, but in this case, it is due to one of their glucopyranose units that is distorted in the ring, forming a truncated cone, leaving these 6 glucopyranose units cyclodextrin with two fewer H-bonds between the C2-OH and C3-OH of different glucopyranose units [13]. These differences in their ability to form inter- and intramolecular H-bonds might explain why some cyclodextrins perform better than others at stabilizing siRNA fragments. Molecular modeling studies of β-cyclodextrins revealed that owing to the secondary belt of H-bonds, water molecules were highly ordered (unfavorable entropy), which suggests that these cyclodextrins could form strong H-bonds with highly ordered -OH structures, such as those of a liposome membrane, or bind to the surface of a nanoparticle. In short, we found that only the seven glucopyranose units cyclodextrins (β-cyclodextrin and methyl-β-cyclodextrin) are good additives for stabilizing siRNAs in liposome formulations. We also observed that these two cyclodextrins stabilize siRNAs in serum by themselves (without liposomes). Furthermore, the data presented here should be useful for future studies on the delivery of siRNA fragments in vivo and in vitro and could serve as a guide for studies aimed at delivering small organic molecules via cyclodextrins, as well as drug solvation or aerosol encapsulation studies [20,21,22].

## 2. Results and Discussion

### 2.1. Serum Stability of siRNA Fragments

As we have previously reported, the serum stability of siRNA fragments are improved when β-cyclodextrins are formulated into liposomes [10]. We extended this study to other cyclodextrin molecules and cyclodextrin-modified nanoparticles. To this end, we encapsulated α, γ and MeβCD with siRNA molecules in DOPC liposomes and incubated these formulations for 48 h at 37 °C in 50% FBS/50% PBS buffer. We used gel electrophoresis to measure the percentage of siRNA remaining intact after a 48 h of incubation period (Figure S1). Table 1**,** entries 1, 2 and 3 show our previous results of the serum stability of siRNAs incubated by themselves, encapsulated in liposomes and encapsulated in liposomes with βCD, respectively, and demonstrated that the addition of βCD to the liposome-siRNA formulation increased the siRNA stability by around 27% [10]. In the present study, however, MeβCD was the best additive tested, helping preserve 91% and 78% of the siRNAs after 24 and 48 h of incubation, respectively (Table 1, entry 4). Alpha and gamma cyclodextrins were the worst stabilizers, and they seemed to disrupt the liposomes after 48 h of incubation (Table 1, entries 5 and 6). The incubation of the siRNA fragments with βCD and MeβCD without liposomes revealed that these cyclodextrins alone stabilized the siRNAs during the 48 h incubation period (Table 1, entries 7 and 8). On the other hand, γ- and α-cyclodextrins had no stabilization effect. Next, we tested gold and selenium nanoparticles modified with β-cyclodextrin molecules on their surface (AuβCD and SeβCD). Compared with their AuβCD counterparts, SeβCD nanoparticles are better at stabilizing siRNAs (Table 1, entries 9 and 10) and better than unmodified β-CD (Table 1, entry 7). As expected, the formulation of these nanoparticle-siRNAs in liposomes did not increase the stability of the siRNAs (Table 1**,** entries 11 and 12). This is probably due to the large size of the AuβCD and SeβCD nanoparticles (around 100 nm in hydrodynamic diameter), which prevents fitting them inside liposomes of around the same size. Finally, to increase the binding of siRNAs to the surface of the SeβCD and AuβCD nanoparticles, we modified the siRNA fragments with an adamantane group (AD) at their 3′ position (siRNA-AD), as adamantane forms strong binding complexes with cyclodextrins [23,24]. We formulated siRNA-AD with SeβCD nanoparticles, with and without a liposome. The results, shown in Table 1, entries 13 and 14, demonstrate that there is a substantial increase in siRNA stabilization, but again, as expected from the size of the nanoparticles (explained above), there was no significant difference in stability between the preparations formulated with and without a liposome. These results show that both β-cyclodextrin and Me-β-cyclodextrin formulated in liposomes improve the stability of siRNA fragments in serum, and interestingly, this siRNA stabilization effect is observed without liposomes. This last finding indicates that carriers consisting of β-cyclodextrin and Me-β-cyclodextrin molecules that do not require complex synthetic steps can be developed as alternatives to conventional nanoparticles for the delivery of siRNA fragments.

### 2.2. Binding Thermodynamics

**Scheme 1:**
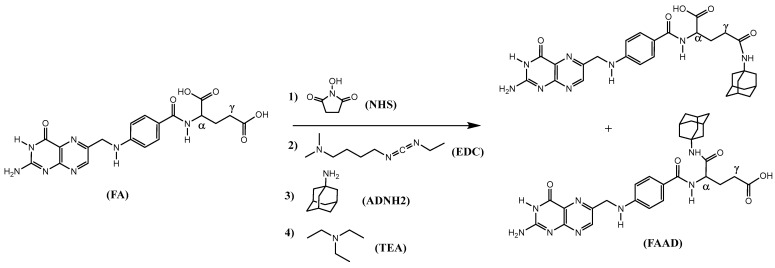
Synthesis of adamantane-folic acid.

**Scheme 2:**
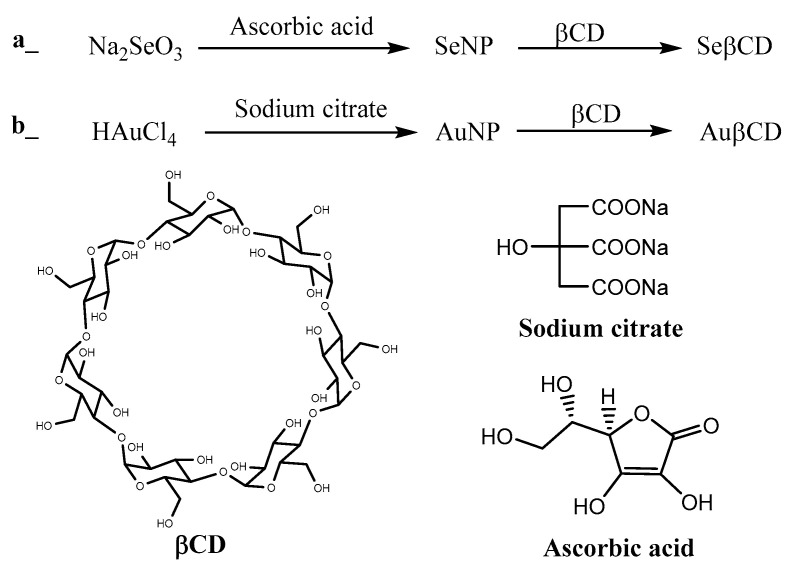
Synthetic scheme of Au and Se nanoparticles. Synthesis of Au and Se nanoparticles starting from their respective salts (**a**,**b**).

**Figure 1:**
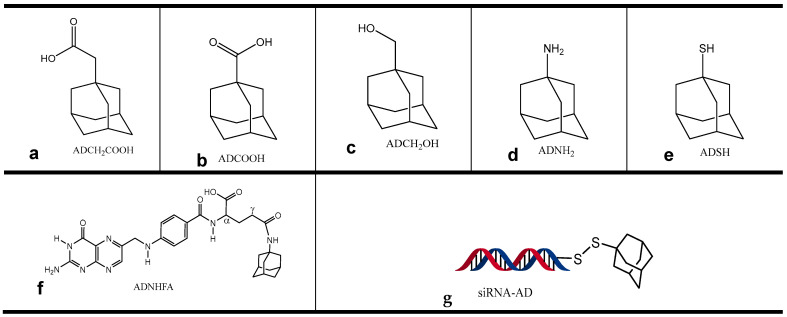
Structures of all adamantane derivatives, folic acid and modified siRNA. Structures of the adamantane derivatives used (upper lane, (**a**–**e**)), folic acid modified with adamantane (lower left, (**f**)), and siRNA modified with adamantane (lower right, (**g**)).

We studied the binding of different adamantane derivatives to cyclodextrins (βCD, MeβCD, αCD, and γCD) and the binding of free and modified siRNA fragments to cyclodextrin-modified selenium and gold nanoparticles (SeβCD and AuβCD) via isothermal titration calorimetry (ITC). These studies allowed us to identify the best adamantane derivatives to modify siRNA molecules and folic acid (FA, commonly used for target delivery to cancerous cells [25]) and to identify the best cyclodextrin molecules to modify nanoparticles (NPs). Table 2 summarizes these results. We calculated the unitless C value (defined in the experimental section, Equations (2a)–(2d)) to ascertain the validity of the data, whereas data that yielded C values ranging from 10 to 500 were considered most reliable [26]. All the adamantane derivatives we studied (ADCH_2_COOH, ADCOOH, ADCH_2_OH, ADNH_2_ and ADSH - shown in Figure 1a–e) displayed strong binding constants (K_A_) to β-cyclodextrin, but ADCOOH, ADSH, and ADCH_2_COOH [10] showed the strongest binding (large K_A_ values, ranging from 3.4 × 10^+5^ to 1.2 × 10^+5^ M^−1^), followed by ADNH_2_ and ADCH_2_OH (K_A_ values of 2.4 × 10^+4^ and 9 × 10^+3^, Table 2, entries 1–5). The enthalpy (ΔH) and free energy (ΔG) of the binding of these adamantane analogs to β-cyclodextrins were similarly favorable (ranging from −26 to −34 KJ/mol and from −30 to −23 KJ/mol, respectively). The entropy contribution, however, was small, suggesting that the interaction was driven by intramolecular attractions rather than the reorientation of water or other molecules [27]. An important aspect in the design of a carrier is to prevent proteins in the cell culture medium from adhering to the nanocarrier surface (forming a protein corona), which changes the physicochemical properties of the nanoparticle, diminishing its delivery properties. However, our results showed that proteins in the medium do not interfere with the binding between the cyclodextrins and the adamantane derivatives. The binding thermodynamics of ADCH_2_COOH to βCD in 50% FBS are not affected by the proteins in the serum (Table 2, entry 6). This suggests that nanoparticles covered with cyclodextrins on their surface are not affected by proteins in the medium. The binding of all the adamantane analogs to the Me-β-cyclodextrins was also strong (large binding constants, K_A_—Table 2, entries 7–11), and ΔH was also favorable. Furthermore, the binding constants we obtained for α- and γ-cyclodextrins to the adamantane derivatives were small (K_A_ < 1000 KJ/mol). These results yielded small C values and therefore the data were not included in this study. To improve the binding of the siRNAs to the cyclodextrin-modified nanoparticles, we chemically bound ADSH (Figure 1e) to the siRNAs via a dithiol bond (siRNA-AD, Figure 1g). Some of the reasons for choosing ADSH are that it strongly binds to β- and Me-β-cyclodextrins (Table 2, entries 3 and 10), and dithiol bonds are reduced inside a cell’s endosome (the most likely route of transfection), releasing the siRNA fragments inside the cell’s cytoplasm. Thus, siRNA chemically modified with ADSH is suitable for in vivo and in vitro experiments. We also modified folic acid (FA), which is commonly used in drug delivery vehicles to target tumorous tissues that overexpress the folate receptor, with an adamantane derivative, ADNH_2_ (Figure 1d,f). We chose ADNH_2_ since it strongly binds to both β- and Me-β-cyclodextrins (Table 2, entries 4 and 11), and the chemical modification of the FA’s γ carboxylic acid group is straightforward (Scheme 1). Finally, we modified the surfaces of selenium and gold nanoparticles with β-cyclodextrins (SeβCD and AuβCD, Scheme 2). β-Cyclodextrin strongly binds to all the adamantane derivatives, and unlike Me-β-cyclodextrin, its -OH groups are free to bind to the metal surface (due to its secondary H-bond belt). However, our results revealed moderate to weak binding constants between siRNA and adamantane-modified siRNA to both selenium and gold nanoparticles modified with β-cyclodextrins (SeβCD and AuβCD). We did not observe a significant difference in binding between the adamantane-modified siRNA (siRNA-AD, Figure 1g) and the unmodified siRNA fragment to the nanoparticles (Table 2, entries 12–15). Notably, the binding constants we observed in these last four experiments are similar to what we previously reported for the binding of β-cyclodextrin to siRNA and for the binding of an adamantane-blocked β-cyclodextrin to siRNA fragments. These surprising results suggest that large molecules such as siRNAs weaken the binding of adamantane groups to cyclodextrins. The entropy contributions of these last 4 binding interactions were more favorable than we observed for all other experiments (hence driven by reorientation of water or siRNA molecules). The binding between AuβCD and folic acid modified with adamantane (ADNHFA, Figure 1f) was also weak, and although the C value fell within the acceptable range, solubility issues forced us to use dilute concentrations of ADNHFAs, which are not ideal; therefore, these results are not very reliable. Nevertheless, folic acid is a large molecule, and it might interfere with the binding of adamantane to cyclodextrin.

The thermodynamic data presented in Table 2 are consistent with data published by other groups and were obtained with similar systems and methods [28]. The enthalpy-driven binding we observed with all the cyclodextrins we studied (entries 1–11) is consistent with predominantly van der Waals interactions between the molecules [27,29,30].

### 2.3. Nanocarrier Synthesis and Characterization

We optimized the synthesis of gold- and selenium–β-cyclodextrin-modified nanocarriers (AuβCD and SeβCD) to obtain homogeneous and stable particles. We achieved this by varying the concentrations of the metal salts, the reducing agents, and the reaction temperature. We routinely measured the hydrodynamic diameter (hd) and surface-charge distribution (Z potential) during the synthesis-optimization steps of the nanocarriers using dynamic light scattering (DLS, Mobious, Wyatt Technology, 6330 Hollister Ave, Santa Barbara, CA 93117, Figures S2–S5). The most homogeneous AuβCD nanocarriers we prepared were around 24 nm (hd) and had a Z potential of around −13 mV, whereas the most homogeneous SeβCD nanocarriers were larger (100 nm and Z = −7 mV). We also determined the stability of the nanocarriers upon storage in aqueous solution for up to 14 days at room temperature via DLS (discussed in the next section), and the results indicate that, during this period, β-cyclodextrin molecules remained attached to the surface of the gold and selenium nanoparticles and that the nanocarriers did not cluster together, forming dimers or larger structures. We purified the final products (AuβCD and SeβCD nanoparticles) via dialysis and determined the concentrations of Au and Se via atomic absorption spectroscopy (AA). We used nuclear magnetic resonance (NMR) spectroscopy to determine the total concentration of βCD, and we measured the hd and Z potential of the final products via DLS. The adamantane derivative oligonucleotide-adamantane (siRNA-AD) was synthesized separately, and the final product was purified via a G25 column.

### 2.4. Nanocarrier Viability and Aqueous Solution Stability Studies

**Figure 2:**
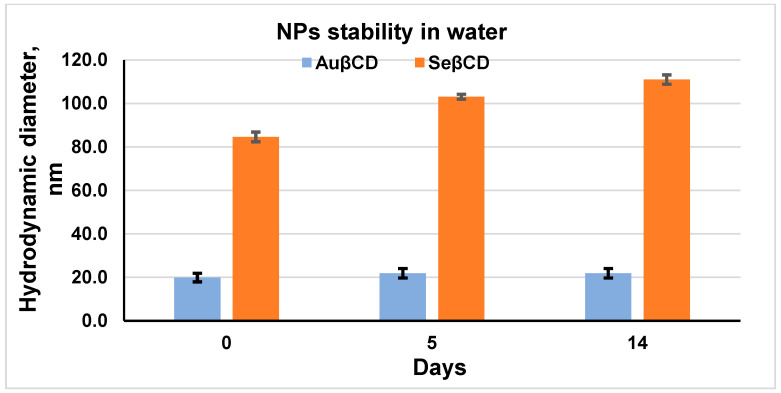
Nanoparticle stability in water. Hydrodynamic diameter of AuβCD and SeβCD nanoparticles stored in H_2_O at room temperature. Error bars represent the ±SD of at least 3 measurements.

**Figure 3:**
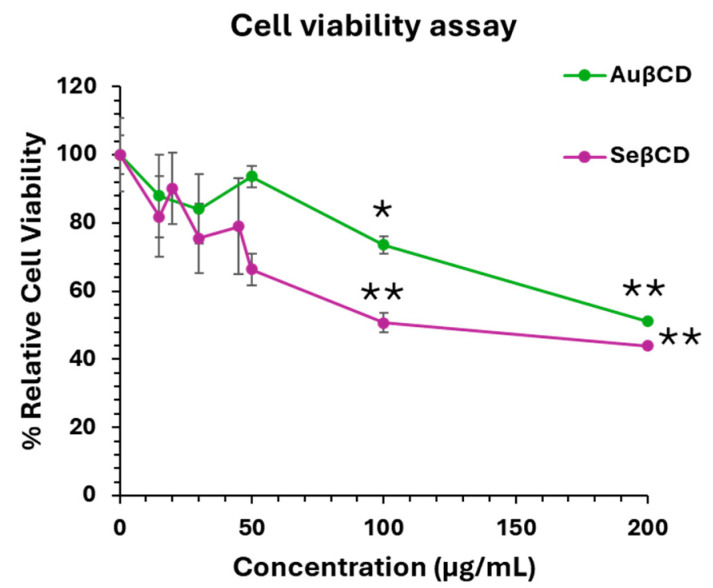
Cell viability studies using the MTS assay. A2780CP20 cells were incubated with different concentration of AuβCD or SeβCD NPs as described in the Materials and Methods section. * *p* < 0.05, ** *p* < 0.01, compared with untreated cells (0.0 μg/mL) of triplicates.

To determine the overall stability of the gold- and selenium-β-cyclodextrin-modified nanocarriers (SeβCD and AuβCD) in aqueous solutions, we incubated them at room temperature (in H_2_O) for up to 14 days and measured their hd and Z potential at 0, 5 and 14 days. We observed that (within the experimental error) the hd of the AuβCD nanocarrier remained constant throughout the incubation period, with an average hd of 21 ± 1 nm. However, we observed a slight increase in the hd of the SeβCD nanocarrier, from 85 ± 2 to 110 ± 2 nm, which could be due to the rearrangement or attachment of free CD molecules in solution. This slight increase in hd could not be due to aggregation of the nanoparticles; otherwise, the size increase would be much larger. Therefore, these results suggest that the nanocarriers do not aggregate into larger complexes or fragment into smaller particles (Figure 2). The Z potential also remained constant during this incubation period (−13 mV for AuβCD and −7 mV for SeβCD). These results also suggest that β-cyclodextrin molecules remain attached to AuβCD and SeβCD during this period in water; otherwise, we would have observed a decrease in hd and a change in the Z potential. In the cell transfection experiments, concentrations of 50 µg/mL or lower of AuβCD or SeβCD did not significantly reduced cell viability (Figure 3). However, concentrations of 100 and 200 µg/mL significantly reduced cell viability. Our Au and Se nanocarriers are covered with βCD molecules, and βCD molecules have been shown to be nontoxic at concentrations of less than 1 mM in HeLa and HEK293T cells lines [31]. These and our results suggest that siRNA could be delivered as AuβCD or SeβCD formulations at concentrations lower than 50 µg/mL. Further mouse models are necessary to test this hypothesis.

Among all NPs, liposomes are the most used delivery systems. In fact, mRNA COVID-19 vaccines are delivered as liposome formulations composed mostly of DOPC, and several anticancer drugs (cisplatin, doxorubicin, and paclitaxel) are also delivered as liposomal formulations [32,33]. Due to their versatility, several AuNPs are also undergoing clinical trials [34]. The unique cyclic oligosaccharide structure of cyclodextrins allows them to form stable inclusion complexes with siRNA, protecting it from enzymatic degradation and facilitating its encapsulation within liposomes. This combination not only improves the pharmacokinetics of siRNA but also enhances its bioavailability by promoting endosomal escape after cellular uptake [35,36]. Recent studies have demonstrated that liposomal formulations utilizing cyclodextrins can significantly increase gene silencing efficiency while minimizing cytotoxicity, making them a valuable tool in RNA-based therapeutics [37]. Thus, the NPs we studied here have the potential as delivery systems not only for siRNA but also for commonly used chemotherapeutic agents. Future experiment using these NPs in vivo models of cancer are envisioned.

## 3. Materials and Methods

Materials. 18:1 (Δ9-Cis) PC (DOPC) 1,2-dioleoyl-snglycero-3-phosphocholine (DOPC), cholesterol, and 1,2-distearoyl-sn-glycero-3-phosphoethanolamine-N-[methoxy- (polyethylene glycol)-2000] ammonium salt (PEG-2000), negative control siRNA (NC-siRNA), β-, Meβ-, γ- and α- cyclodextrins, all adamantane derivatives, folic acid and all solvents were purchased from Sigma Aldrich (St. Lois, MO, USA).

### 3.1. Optimization of the Synthesis of SeβCD and AuβCD Nanoparticles

To obtain highly monodispersed nanoparticles [low polydispersity index (PDI)] with a hydrodynamic diameter (hd) in the range of 20−200 nm, we varied the concentrations of the reducing agents, metal salts, β-cyclodextrin and temperature to characterize the nanoparticles obtained after each step via dynamic light scattering (DLS, Figures S2–S5 and Tables S1–S6). Scheme 2 outlines the synthesis of both selenium and gold nanoparticles modified with β-cyclodextrin (SeβCD and AuβCD). The first parameter we changed was the concentration of the reducing agent while keeping the concentration of the metal salt constant. After choosing the optimum concentration of reducing agent, we optimized the concentration of the metal salt (the reducing agent concentration was kept constant with that found in the previous step,). Following a similar procedure and keeping the concentrations of both the reducing agent and the metal salt constant (using the optimum concentrations identified previously), we proceeded to optimize the temperature and, in a similar fashion, the concentration of β-cyclodextrin. To optimize the selenium nanoparticles (SeNPs), we reacted them with 4.0, 8.0- and 12.0-mM ascorbic acid while keeping the concentration of Na_2_SeO_3_ constant at 2.0 mM (reaction at 25 °C). The best result was obtained with 8.0 mM of the reducing agent ascorbic acid, which yielded homogeneous SeNPs with an hd of less than 200 nm, a Z potential of −35 ± 2 mV, and a PDI of 0.03 ± 0.02 (Table S1, Figure S2). The other two concentrations used resulted in similar sizes and charge distributions of SeNPs, except that their PDI was greater (Table S1). Next, we reacted with 1.0, 2.0, and 4.0 mM Na_2_SeO_3_ while keeping the ascorbic acid concentration constant at 8.0 mM at 25 °C. The best result was obtained with the original concentration of Na_2_SeO_3_ (2.0 mM; Table S2, Figure S3). Reacting 1.0 mM Na_2_SeO_3_ resulted in NPs larger than 200 nm in hd. At this point, we optimized the reaction temperature, repeating the reaction (using the optimum concentrations of ascorbic acid and Na_2_SeO_3_ determined in the previous steps) at 60 °C and 80 °C. We found that 25 °C (the original reaction temperature) was the best reaction temperature since the SeNP hd and heterogeneity (more than one peak was observed via DLS) increased with temperature. The zeta potential and PDI were not affected by the reaction temperature. The next step of SeNP synthesis involves attaching β-cyclodextrin molecules to its surface (Table S3). Of the two β-cyclodextrin concentrations used, 1.0 and 8.0 mM, the first concentration (1.0 mM βCD) resulted in smaller but nonhomogeneous NPs (153 ± 2 nm hd), whereas the last concentration yielded homogeneous SeβCD particles of 195 ± 2 nm in hd, with a zeta potential of −7 ± 1 mV and a PDI = 0.17 ± 0.04; therefore, this concentration was the concentration of β-cyclodextrin used for all the experiments. NMR studies (discussed in a subsequent section) revealed 84% β-cyclodextrins on the NP surface.

The gold nanoparticles (Au NPs) were optimized following a similar protocol (Scheme 2). First, we optimized the concentration of the reducing agent (sodium citrate) while maintaining the concentration of HAuCl_4_ constant at 2.0 mM (Table S4 and Figure S4 of DLS analysis). Reacting with 2.0, 8.0- and 12.0-mM sodium citrate at 25 °C yielded AuNPs of similar size and zeta potential; however, with 8.0 mM, we obtained the most homogeneous Au particles, with a PDI of 0.24 ± 0.01, a zeta potential of −13.2 ± 0.4 mV, and an hd of 24 ± 1 nm (Table S4). Lowering the concentration of HAuCl_4_ from 2.0 mM to 1.0 mM while keeping the concentration of sodium citrate constant (at 8.0 mM) did not have a major effect on the characteristics of the AuNPs; however, increasing the concentration to 4.0 mM HAuCl_4_ resulted in nanoparticles larger than 200 nm in hd (Table S5 and Figure S5 of DLS analysis). Therefore, in subsequent reactions, we used 2.0 mM HAuCl_4_ and 8.0 mM sodium citrate. Next, we optimized the concentration of βCD (Table S6). Reacting with 4.0, 8.0, 12.0 or 16.0 mM βCD did not have a major effect on the AuβCD nanoparticle size or zeta potential, yielding NPs in the range of 20 ± 2 to 25 ± 2 nm in hd and from −20 ± 2 to −37 ± 2 mV (zeta potential). However, 12.0 mM βCD yielded homogeneous NPs, and NMR spectroscopy revealed 86% wt/wt β-cyclodextrin molecules; therefore, this concentration was used (Table S6).

### 3.2. Optimized Synthesis of β-Cyclodextrin-Modified Selenium Nanoparticles (SeβCD)

**Figure 4:**
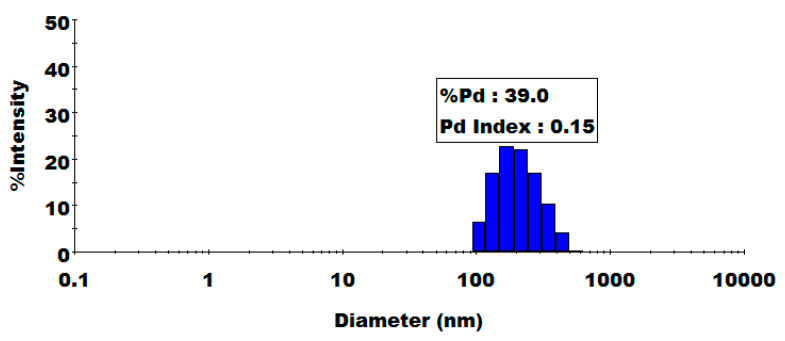
DLS analysis of the optimized SeβCD nanoparticles in water. Following the optimizing procedures, the hydrodynamic diameter of SeβCD nanoparticles was measured by Dynamic Light Scattering (DLS).

An amount of 4 mL of an ascorbic acid aqueous solution (ascorbic acid, 0.2 mmol) was added dropwise to a 6 mL Na_2_SeO_3_ solution (0.05 mmol) and allowed to react with stirring for 30 min to form selenium nanoparticles (SeNPs). The SeβCD nanoparticles were synthesized by adding 15 mL of a β-cyclodextrin aqueous solution (βCD, 0.2 mmol) dropwise to the SeNP solution and reacting for 2 h with stirring at room temperature (Scheme 2a). The final product was dialyzed overnight against an excess of deionized water (MWCO 12–14 kDa). The DLS results for these nanoparticles in water are shown in Figure 4. The optimized nanoparticles were used for all subsequent studies.

### 3.3. Optimized Synthesis of β-Cyclodextrin-Modified Gold Nanoparticles (AuβCD)

**Figure 5:**
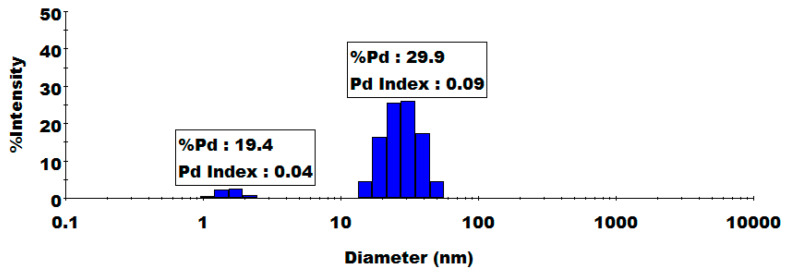
DLS analysis of the optimized AuβCD nanoparticles in water. Hydrodynamic diameter of AuβCD nanoparticles measured by Dynamic Light Scattering (DLS).

A total of 10 mL of sodium citrate aqueous solution (0.4 mmol) was added dropwise to 40 mL of HAuCl_4_ solution (0.1 mmol) with stirring for 2 h to form gold nanoparticles (AuNPs). The final AuβCD nanoparticles were synthesized by adding 20 mL of a β-cyclodextrin aqueous solution (βCD, 0.6 mmol) dropwise to the AuNP solution, and the mixture was allowed to react with stirring for 2 h (Scheme 2b). The product was subsequently dialyzed overnight against an excess of deionized water (MWCO 12–14 kDa). The DLS results for these nanoparticles in water are shown in Figure 5. The optimized nanoparticles were used for all subsequent studies.

### 3.4. Characterization and Water Stability of SeβCD and AuβCD Nanoparticles by DLS

The size and charge distribution of the selenium and gold nanoparticles modified with β-cyclodextrins (SeβCD and AuβCD) were determined via dynamic light scattering (DLS). The particle size and zeta potential of the samples in a water solution were measured after 5 and 14 days of incubation at room temperature with a Mobius instrument (Wyatt Technology). The Mobius instrument measures the zeta potential and the particle hydrodynamic diameter (hd) simultaneously, using a unique design that allows a current to be drawn between two electrodes inside a 45 µL cell. As particles migrate from one electrode to the other, a laser beam passes between the electrodes, and its diffraction pattern (from encountering the particles) is captured by 31 detectors arranged 5 degrees from each other. This arrangement allows us to accurately measure the particle size and charge distribution.

### 3.5. Determination of the βCD Concentrations on the AuβCD and SeβCD Surfaces via NMR Spectroscopy

The concentrations of the β-cyclodextrin molecules appended onto the surface of the Au and Se nanoparticle cores were determined via NMR spectroscopy via a calibration curve method as previously described by Zhao et al. with some modifications [38]. A calibration curve was obtained by plotting the ratios of the areas under the peaks at 355 ppm and 2.15 ppm, corresponding to β-cyclodextrin and acetone-d6 (used at small concentrations as an internal standard), versus the β-cyclodextrin concentration of β-cyclodextrin/deuterated water (D_2_O) solutions, with gradient β-cyclodextrin concentrations ranging from 0.08 to 0.8 mM. Deuterated acetone (17% *v*/*v*—kept constant throughout the solutions) was used as an internal standard. To determine the concentration of appended β-cyclodextrin, D_2_O solutions of the lyophilized powders of AuβCD and SeβCD were prepared (0.5 mg/mL, containing 17% *v*/*v* acetone-d6), and their H^1^ NMR data were recorded. The concentration of β-cyclodextrin was then determined via a calibration curve, and the ratios of the areas under the peaks at 3.55 ppm and 2.15 ppm were obtained for the AuβCD and SeβCD solutions.

### 3.6. Determination of the Au and Se Concentrations in the AuβCD and SeβCD Nanoparticles via Atomic Absorption Spectroscopy (AA)

After the nanoparticles were synthesized, the concentrations of the metals were determined via AA spectroscopy (PerkinElmer AAnalyst 200, with acetylene used as fuel and dedicated Lumina hollow cathode lamps) from calibration curves obtained via standard aqueous solutions of Se and Au. A calibration curve was obtained by plotting the absorbance versus the Se or Au concentration of the corresponding standard aqueous solutions (PerkinElmer standard solutions), with gradient concentrations ranging from 1 to 25 ppm for Au and 10 to 45 ppm for Se. In theory, knowing the concentration of the metals and the hydrodynamic diameter (hd) of the particles, it should be possible to calculate the concentration of nanoparticles in solution and therefore the total surface area available to β-cyclodextrin molecules. Since we know the concentration of β-cyclodextrin molecules in the solutions (obtained via NMR), it should be possible to estimate the number of β-cyclodextrin molecules on the nanoparticle surface. Unfortunately, without knowing the exact shape and physical characteristics of the nanoparticles (if they are solid or hollow—Au nanoparticles have been reported to be hollow), this exercise would lead us to approximations that could be quite far from reality; therefore, we decided not to speculate as to the number of cyclodextrin molecules on the surface of the nanocarriers but simply consider their total concentration in the solution of nanoparticles, as determined by NMR spectroscopy.

### 3.7. Cell Culture and Drug Treatment

The A2780CP20 cells were provided by Dr. Anil K. Sood (MD Anderson Cancer Center, Houston, TX, USA) and have been described elsewhere [2,3]. For propagation, the cells were maintained in RPMI-1640 medium (Thermo Scientific, Logan, UT, USA) supplemented with 10% fetal bovine serum (FBS) (Thermo Scientific, Logan, UT, USA) and 0.1% antibiotic/antimycotic solution (Thermo Scientific, Logan, UT, USA). All the cells were maintained at 37 °C in 5% CO_2_ and 95% air. The cell lines were screened for mycoplasma via the LookOut® Mycoplasma PCR detection kit as described by the manufacturer (Sigma-Aldrich, St. Louis, MO, USA) and authenticated by Promega (Madison, WI, USA) and ATCC via short tandem repeat (STR) analysis. All in vitro assays were performed at a 70–85% cell density. For cell viability, cells (2 × 10^4^ cells/mL) were seeded into 96-well plates. Twenty-four hours later, the cells were exposed to the nanocarriers. Seventy-two hours later, the % cell viability of the nanocarriers (SeβCD and AuβCD) was determined using the MTS assay. Optical density (OD) values were obtained via a plate reader (Bio-Rad), and percentages of cell viability were calculated after blank OD subtraction; the untreated cell values were considered 100% cell viability. The values are expressed relative to those of untreated cells, which were considered 100% cell viability. In all the cases, we found that, up to the maximum concentration used (200 µg/mL), the % cell viability did not fall below 50% (Figure 3). Typically, around 1.5 µg/mL of siRNA, and 3 µg/mL of SeβCD or AuβCD nanoparticles are used for transfection experiments - well below the nanoparticle concentration used in these MTS assays.

### 3.8. Liposome Preparation

DOPC (0.1 mg) was mixed with cholesterol (50% *w*/*w* DOPC) and PEG-2000 (50% *w*/*w* DOPC) in excess of tert-butanol. The mixture was frozen in an acetone-dry ice bath and lyophilized. Then, the siRNA (10 μg) was mixed with the corresponding cyclodextrin (1:30 *w*/*w* siRNA:βCD) in the presence of Ca^2+^ and Mg^2+^-free PBS and incubated for 15 min. The siRNA/CD complex was added to the lyophilized liposome mixture, vortexed for 2 min, and sonicated for 15 min.

### 3.9. Serum Stability Measurements

For serum stability, liposomes (containing siRNA/CD) were incubated at 37 °C in 300 μL of 50% FBS in PBS buffer, pH 7.4. Aliquots of 50 μL were withdrawn at 0, 24, and 48 h and frozen at −20 °C. The loading dye (5 μL) was added to the samples (15 μL) and then loaded into a 3% tris-borate-ethylenediaminetetraacetic acid (TBE) agarose gel (1% EtBr, Figure S1.

### 3.10. Thermodynamic Studies

**Figure 6:**
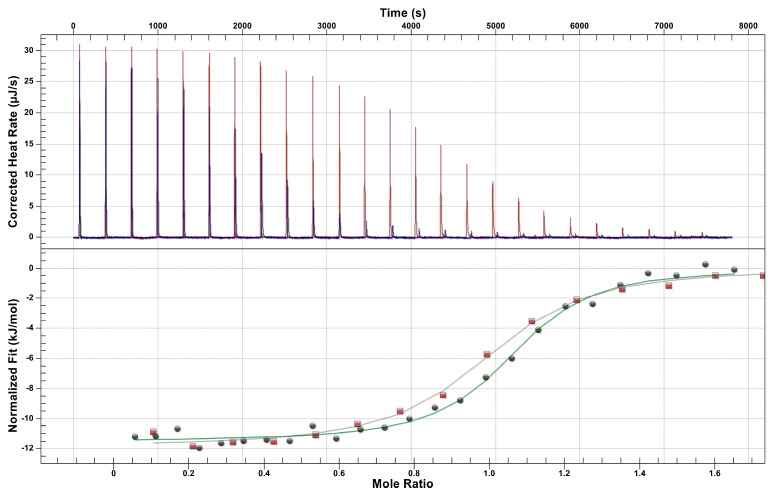
Thermogram of binding between β-cyclodextrins and 1-adamantane acetic acid. Isothermal titration calorimetry. Upper: thermogram obtained from injecting βCD (red) and MeβCD (blue) into ADCH_2_COOH. Lower: fitting curves of the areas obtained from the thermograms shown in the upper part of the graph. Red squares: MeβCD + ADCH_2_COOH; dark circles: βCD + ADCH_2_COOH.

Isothermal titration calorimetry (ITC, Affinity, TA instruments) was used to study the binding thermodynamics between β-, methyl-β-, γ-, and α-cyclodextrins (βCD, MeβCD, γCD, αCD), and selenium and gold nanoparticles modified with β-cyclodextrin (SeβCD and AuβCD) to different adamantane derivatives and siRNAs (Table 2). Aqueous solutions of βCD, MeβCD, γCD, αCD, SeβCD and AuβCD nanoparticles (typically, 2–5 µL of 3–6 mM) were titrated against the adamantane derivatives, injected at 300 sec intervals into a solution of the corresponding adamantane derivatives, placed in the sample cell of the instrument (0.3–0.6 mM in water), and stirred at 150 r.p.m. at 25 °C. The reference cell contained water alone. The amount of heat produced per injection was calculated by integrating the areas under each individual peak via instrument software (NanoAnalyze^TM^ software, v 3.4.0, TA instruments), taking into consideration the heat of dilution. The experimental data were fitted to a theoretical titration curve provided by NanoAnalyze^TM^ software, in which the independent variables of interest, ΔH, the enthalpy change in kJ mol^−1^, K_A_, the association constant in M^−1^, and n, and the complex stoichiometry, were calculated via the “independent binding sites” model as described in the literature [39]. K_A_ is obtained from the slope of the inflection point at mid-titration of a graph of the heat liberated (or consumed), normalized to the concentration in the syringe, vs the injection # or mole ratio (Figure 6, lower graph). ΔH is obtained from the largest heat released or consumed (usually during the first injections), normalized to the concentration in the syringe (Figure 6, upper thermogram). The stoichiometry (n) is obtained from the mole ratio of the reactants at the inflection point of the titration graph (Figure 6, lower graph). Note that both K_A_ and ΔH are affected by the concentration in the syringe, but neither is affected by the concentration in the cell, whereas n is affected by both concentrations (in the cell and in the syringe). ΔS and ΔG are dependent variables calculated via Equations (1a) and (1b).

ΔG = −RTlnK_A_ (1a)

ΔG = ΔH − TΔS(1b)

The unitless C value is used to determine the validity of the results (binding and dissociation constants K_D_ and K_A_ and the enthalpy and free energy, ΔH and ΔG), and it is calculated from Equations (2a)–(2d):

(2a) $$C=N\frac{\left[M\right]}{KD}$$

where:

[M] concentration in the cell

N = binding ratio of macromolecules:

(2b) $$N=St\frac{Afcell}{Afsyringe}$$

(2c) $$Afcell=\frac{\left[active\;molecule\right]}{\left[total\;molecule\right]}$$

(2d) $$Afsyringe=\frac{\left[active\;molecule\right]}{\left[total\;molecule\right]}$$

St = Stoichiometry

To estimate the C values, we adjusted the concentration in the cell so that n = 1 (we assumed that the stoichiometry of AD derivatives to CDs is 1 and that all molecules should be “active”—equations b–d). C values between 1 and 500 are considered acceptable for extracting K_A_. (although C values between 10 and 100 are the best for extracting K_A_). C values less than 1 or greater than 1000 are not reliable and are usually rejected. Two examples of the data obtained from the binding between ADCH_2_COOH and βCD and MeβCD are shown in Figure 6.

### 3.11. Synthesis of Adamantane Derivatives

**Scheme 3:**
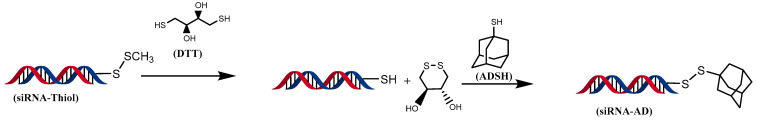
Synthesis of AD-siRNA.

Synthesis of oligonucleotides-*AD*. The modified oligonucleotide of choice (siRNA)—thiolated at position C3 with and without a cyanine 5 dye (Cy5)—was synthesized as follows: SiRNA-Cy5-Thiol (2.5 nmol) was mixed with 100 µL (10 µmol, solution in 0.15 M phosphate buffer at pH 8.4) dithiothreitol (DTT) and reacted for 1 h in the dark with shaking at room temperature. After this time, the mixture was passed through a G25-5 mL column, and deionized water was used as the solvent to remove unreacted DTT and HSCH_3_ (Scheme 3). After the column death volume was eluted (1.2 mL—fraction #1), the next 1.5 mL (fraction #2) was collected and placed in a 5 mL vial. Adamantane thiol (ADSH) was added (7 mg/20 µL tert-butyl alcohol, 0.042 mmol), and the mixture was allowed to react for 24 h with shaking at room temperature. After the reaction was completed, the mixture was passed through a G25–5 mL column, and the products of the second and third fractions (1.0 mL fractions) were collected. The final product was lyophilized and used without further purification. The product was washed with tert-butyl alcohol and then lyophilized to sterilize it for in vitro studies.

Synthesis of folic acid–adamantane (FAAD). Folic acid (FA, 100–0.23 mmol) was dissolved in 8 mL of anhydrous dimethyl sulfoxide (DMSO) in a round-bottom flask under argon gas. Then, N-(3-dimethylaminopropyl)-N′-ethylcarbodiimide hydrochloride (EDC), 43 mg (0.23 mmol), N-hydroxysuccinimide (NHS), and 26 mg (0.23 mmol) were added to the FA mixture and allowed to react for 1 h under argon gas at room temperature with stirring in the dark (Scheme 1). A solution of adamantylamine (ADNH_2_, 34 mg, 0.23 mmol) and trimethyl amine (TEA, 0.033 mL, 0.23 mmol) in 2.0 mL anhydrous DMSO under argon gas was added. The mixture was left to react overnight at room temperature with stirring and in the dark. The DMSO was then removed by roto evaporation, and the yellow residue of the product was washed several times with tetrahydrofuran (THF) and dried under vacuum. H^1^ NMR spectroscopy revealed the crude product (approximately 85% purity), which was used without further purification.

## 4. Conclusions

Liposomes are excellent delivery vehicles for siRNAs for in vitro and in vivo applications. The retention of siRNAs inside liposomes can be improved by combining the siRNA cargo with Me-β-cyclodextrin or β-cyclodextrin. The optimum ratio of siRNA to cyclodextrins and the possible depletion effect of cholesterol by cyclodextrins need further study. SeβCD and AuβCD nanoparticles provide siRNA fragments adequate protection from degradation in serum (stabilization), but a greater effect is observed when the siRNA fragments are chemically modified with an adamantane group (siRNA-AD). However, our thermodynamic data revealed that neither the siRNAs nor the adamantane-modified siRNAs (siRNA-AD) formed strong complexes with the SeβCD and AuβCD nanoparticles. A possible explanation for this discrepancy is that siRNA molecules might bind sideways along the surface of the nanoparticles. This would partially block nucleases in the medium from digesting the siRNA fragments. Our results with folic acid (FA, commonly incorporated in nanocarrier formulations for cell targeting, particularly for ovarian cancer and other tumor types that express high amounts of folate receptors), modified with adamantane (FA-AD), were not encouraging. The ITC data revealed weak binding between FA-AD and AuβCD, which we suspect was due to the size of folic acid and perhaps the position of substitution of the adamantane group in the FA molecule.

However, our results reveal that the binding constants between all the adamantane derivatives to βCD and MeβCD approach those of protein inhibitors (K_D_ in the range of 10^−5^–10^−7^ M). In our search for adamantane molecules to modify siRNAs and FAs, we collected binding thermodynamic data between cyclodextrins and several adamantane derivatives, which could serve as a foundation for studies aimed at using cyclodextrins for delivery vehicles and solvation or aerosol encapsulation of small organic molecules. The large binding constant (low dissociation constant, K_D_) exhibited by cyclodextrins with small organic molecules might be an undesirable factor that should be considered in drug delivery systems, where the drug cargo must be released inside the cytoplasm of a cell. However, further studies on the effects that the pH and other molecules present in serum (such as cholesterol) might have on the K_D_ of cyclodextrin-drug systems are needed. The results presented here highlight the potential of β- and Me-β-cyclodextrins for the delivery of siRNA fragments in vitro and in vivo in formulations with and without liposomes and with metal nanoparticles. Formulations using liposomes or metal nanoparticles have the advantage of being able to incorporate a targeting moiety (for targeted delivery).

## Figures and Tables

**Table 1:** Serum stability of the siRNA fragments.

Type of siRNA	With or without Liposome Encapsulation	Additive	% siRNA Remaining in the Gel after the Following Incubation Time		Entry
			24 h	48 h	
NC siRNA	No liposome	* None	3	0	1
	Liposome	* None	44 ± 4 **	32 ± 3	2
		* βCD	56 ± 6	42 ± 4	3
		MeβCD	91 ± 9	78 ± 8	4
		αCD	76 ± 8	9 ± 1	5
		γCD	52 ± 5	15 ± 2	6
	No liposome	βCD	38 ± 4	45 ± 4	7
		MeβCD	80 ± 8	30 ± 3	8
		SeβCD	67 ± 7	35 ± 4	9
		AuβCD	18 ± 2	21 ± 2	10
	Liposome	SeβCD	63 ± 6	43 ± 4	11
		AuβCD	15 ± 2	20 ± 2	12
NC siRNA-AD	No liposome	SeβCD	80 ± 8	79 ± 8	13
	Liposome	SeβCD	95 ± 10	98 ± 10	14

The results were obtained from incubation experiments in 50% FBS and analyzed by gel electrophoresis. * Castillo et al. [10]. ** Errors were calculated from 3 measurements.

**Table 2:** Binding thermodynamic results between various cyclodextrins and adamantane analogs and siRNA fragments.

Entry	Syringe	Cell	K_A_ (M^−1^)	ΔH (kJ/mol)	TΔS (kJ/mol·K)	ΔG (kJ/mol)	K_D_ (M)	C
1	βCD *	ADCH_2_COOH	1.2 × 10^5^	−26	0.015	−30	5.4 × 10^−6^	55
2	βCD	ADCOOH	3.4 × 10^5^	−34	−0.007	−32	3 × 10^−6^	64
3	βCD	ADSH	2.2 × 10^5^	−25	0.018	−30	4.5 × 10^−6^	15
4	βCD	ADNH_2_	2.4 × 10^4^	−19	0.020	−25	4.1 × 10^−5^	10
5	βCD	ADCH_2_OH	9.0 × 10^3^	−30	−0.026	−23	1.1 × 10^−4^	7
6	βCD/in serum	ADCH_2_COOH/ in serum	1.2 × 10^5^	−20	0.043	−29	8.4 × 10^−6^	50
7	MeβCD	ADCH_2_COOH	7.8 × 10^4^	−14	0.022	−28	1.3 × 10^−5^	27
8	MeβCD	ADCOOH	2.2 × 10^5^	−24	0.051	−30	4.5 × 10^−6^	69
9	MeβCD	ADCH_2_OH	6.9 × 10^4^	−13	0.049	−28	1.4 × 10^−5^	36
10	MeβCD	ADSH	1.1 × 10^5^	−14	0.056	−29	9.5 × 10^−6^	11
11	MeβCD	ADNH_2_	1.3 × 10^4^	−7	0.015	−23	7.7 × 10^−5^	8
12	AuβCD	siRNA	5.5 × 10^3^	−10	11.0	−21	1.8 × 10^−4^	5
13	SeβCD	siRNA	1.7 × 10^2^	−2.2	10.6	−13	5.7 × 10^−3^	181
14	AuβCD	siRNA-AD	4.8 × 10^3^	−7.5	13.6	−21	2.5 × 10^−4^	159
15	SeβCD	siRNA-AD	1.0 × 10^3^	−2.8	14.5	−17	9.6 × 10^−4^	34
16	AuβCD	ADNHFA	1.0 × 10^3^	−28	0.000	−17	1.0 × 10^−3^	33

Binding thermodynamic data obtained using an ITC instrument (Affinity, TA instruments, New Castle, DE, USA). Most experiments were performed in triplicate, and the errors ranged from 1 to 9%. * Castillo et al. [10].

## Data Availability

The authors confirm that the data supporting the findings of this study are available within the article and its Supplementary Materials.

## Supplementary Materials

The following supporting information can be downloaded at: https://www.mdpi.com/article/10.3390/ph17101344/s1, Figure S1: Agarose gel electrophoresis of siRNAs. Figure S2: DLS result of the optimum concentration (solution highlighted on Table S1). Figure S3: DLS result of the optimum concentration (solution highlighted on Table S2). Figure S4: DLS result of the optimum concentration (solution highlighted on Table S4). Figure S5: DLS result of the optimum concentration (solution highlighted on Table S5). Table S1: Determining the optimal concentration of the reducing agent, ascorbic acid (Vc) for the synthesis of selenium nanoparticles. Table S2: Determining the optimal concentration of selenium salt for the synthesis of selenium nanoparticles. Table S3: Determining the optimal concentration of βCD for the synthesis of cyclodextrin-modified selenium nanoparticles. Table S4: Determining the optimal concentration of the reducing agent, sodium citrate (Ct) for the synthesis of gold nanoparticles. Table S5: Determining the optimal concentration of gold salt for the synthesis of gold nanoparticles. Table S6: Determining the optimal concentration of βCD for the synthesis of cyclodextrin-modified gold nanoparticles.
