# Supplementary material for: Effect of Cyclodextrins Formulated in Liposomes and Gold and Selenium Nanoparticles on siRNA Stability in Cell Culture Medium
Source: Pharmaceuticals (Basel). 2024 Oct 8;17(10):1344. doi: 10.3390/ph17101344 (PMC11510567; doi:10.3390/ph17101344)
Supplement: Supplementary file 1 [file pharmaceuticals-17-01344-s001.zip › pharmaceuticals-3172809-supplementary.pdf]

Effect of cyclodextrins formulated in liposomes and gold and selenium nanoparticles on the siRNA stability in cell culture medium

<u>Supplemental Data</u>

<u>Figure S1</u>: Agarose gels electrophoresis of siRNAs. All gels were run in 50% FBS and PBS (pH 7.2) - all gels have NC siRNA (when not specified that AD-siRNA was used instead).

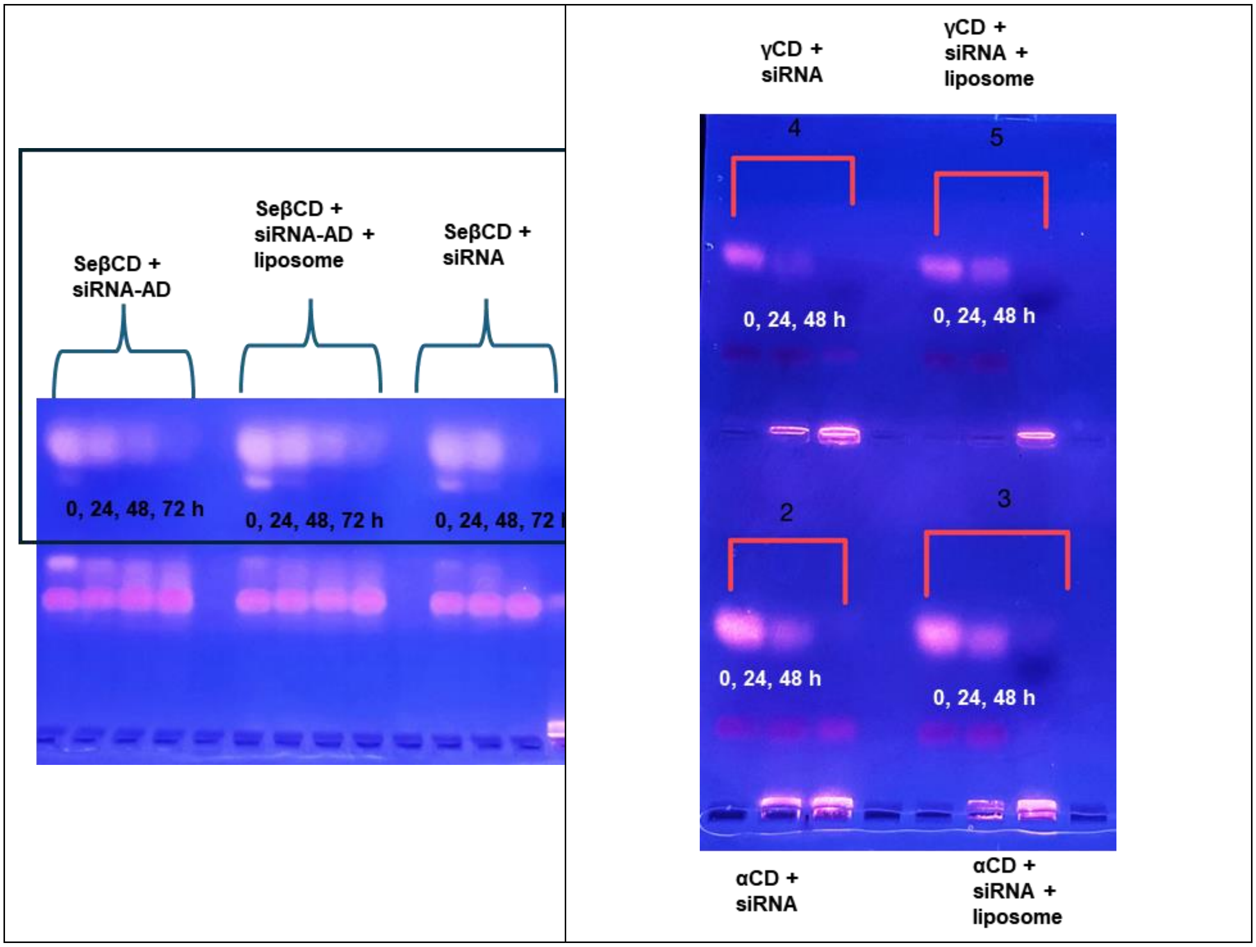

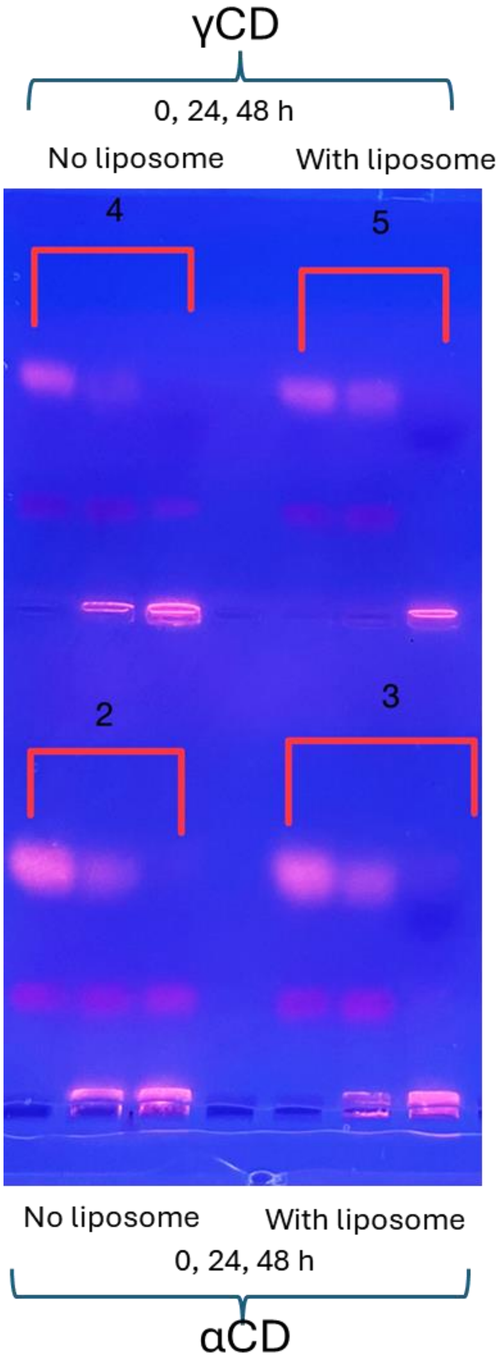
γCD
0, 24, 48 h
No liposome
With liposome
4
5
2
3
No liposome
With liposome
0, 24, 48 h
αCD

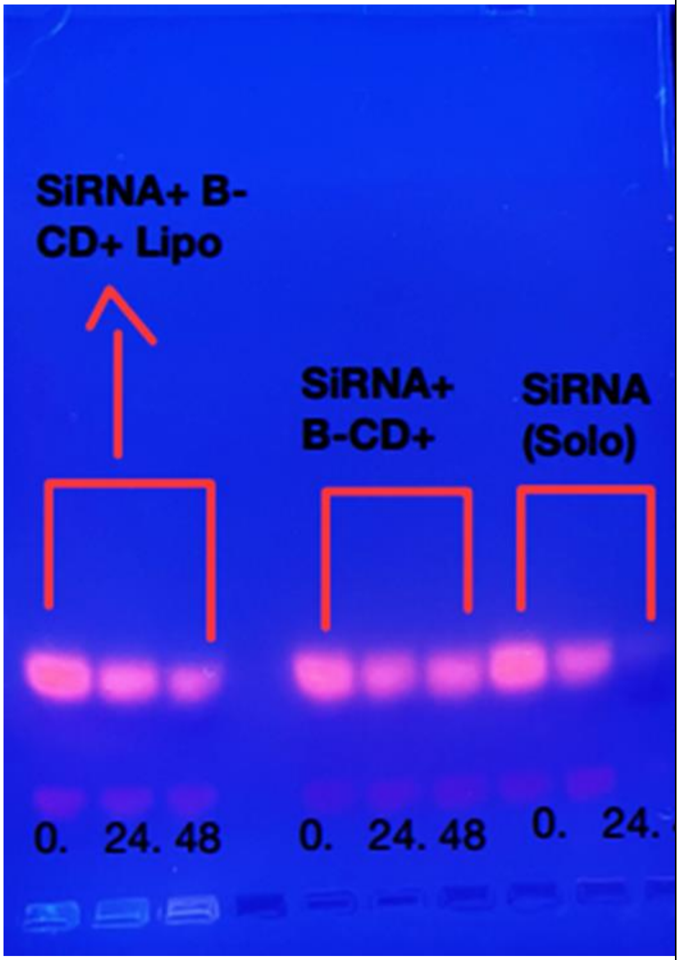
SiRNA+ B-CD+ Lipo
SiRNA+ B-CD+
SiRNA (Solo)
0. 24. 48
0. 24. 48
0. 24.

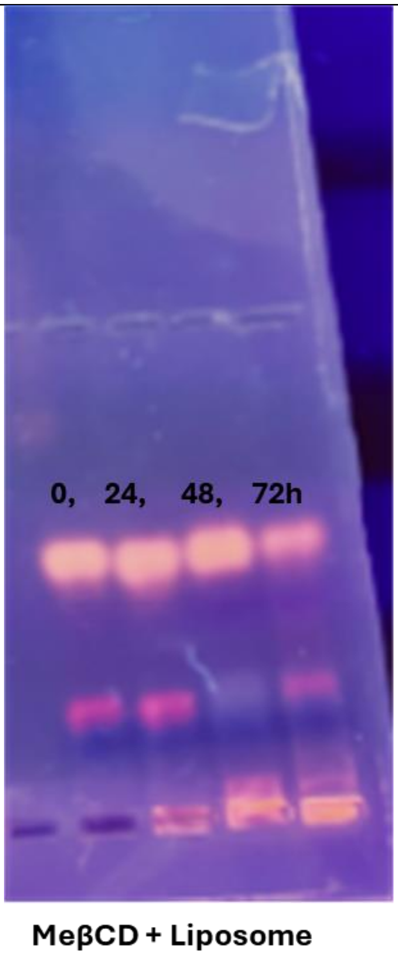
0, 24, 48, 72h
MeβCD + Liposome

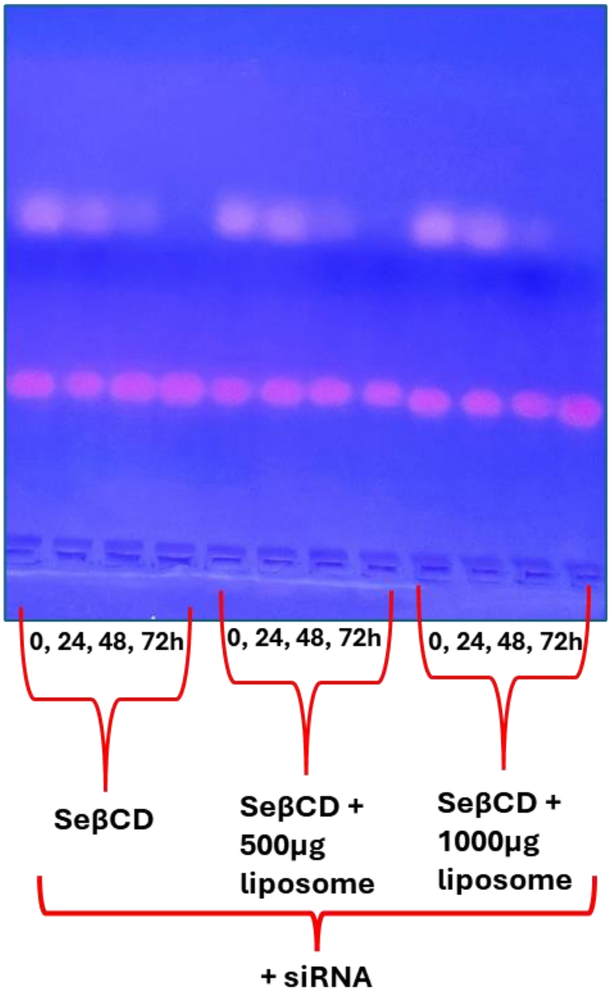
0, 24, 48, 72h
0, 24, 48, 72h
0, 24, 48, 72h
SeβCD
SeβCD + 500µg liposome
SeβCD + 1000µg liposome
+ siRNA

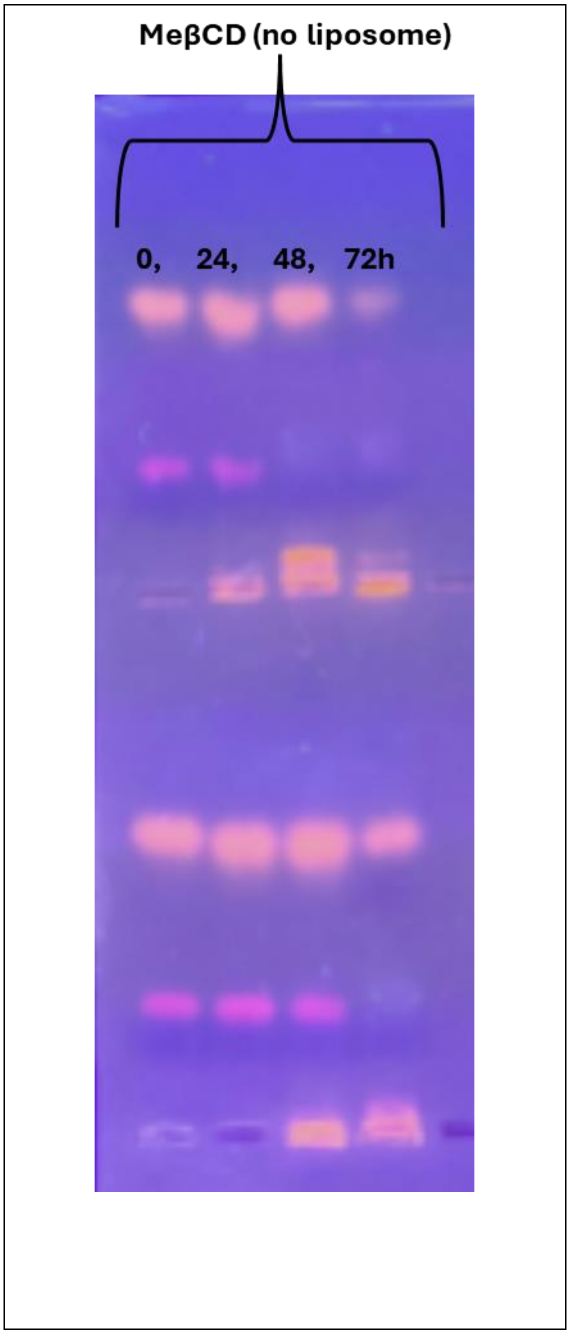
MeβCD (no liposome)
0, 24, 48, 72h

Figure S2: DLS result of the optimum concentration (solution- -highlighted on Table S1)

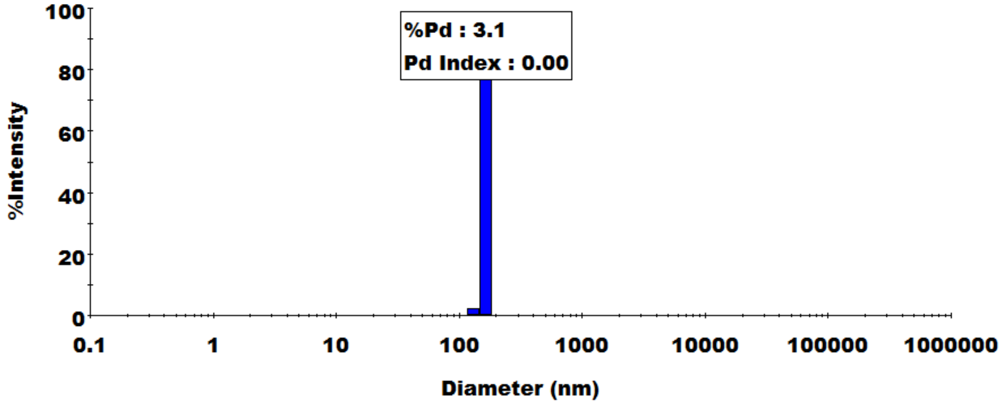

Figure S3: DLS result of the optimum concentration (solution --highlighted on Table S2)

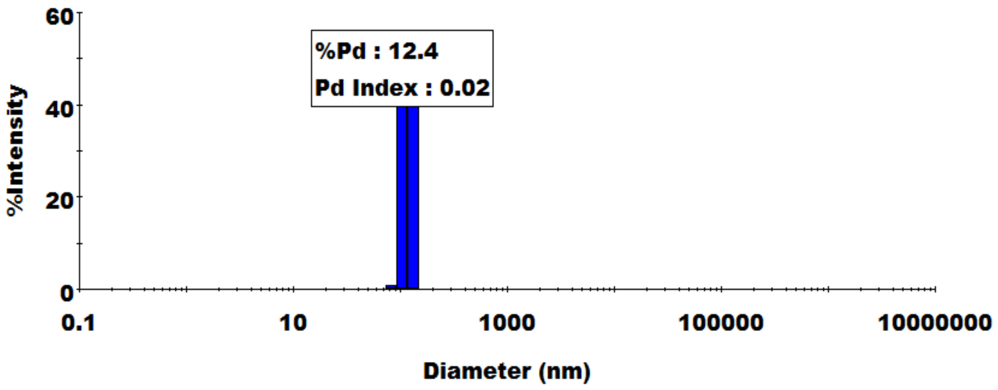

Figure S4: DLS result of the optimum concentration (solution --highlighted on Table S4)

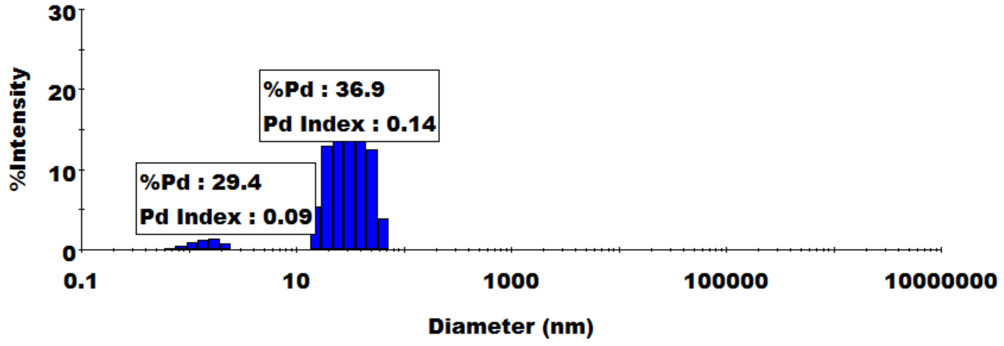

Figure S5: DLS result of the optimum concentration (solution– highlighted on Table S5)

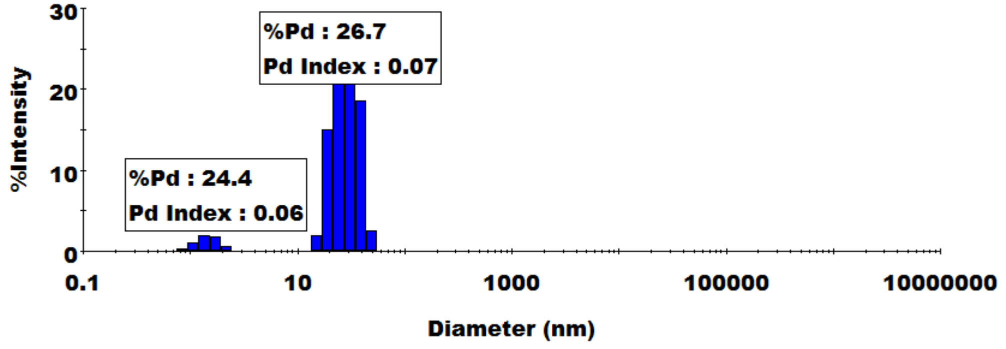

Table S1: Determining the optimal concentration of the reducing agent, ascorbic acid (Vc) for the synthesis of selenium nanoparticles

mM Vc	Diameter (nm)	SD	Zeta Potential	SD	Se:Vc ratio
4	177	4	-24	1	1:2
**8**	169	1	-35	1	**1:4**
12	162	1	-28	1	1:6

Table S2: Determining the optimal concentration of selenium salt for the synthesis of selenium nanoparticles

mM Na_2SeO_3	Diameter (nm)	SD	Zeta Potential	SD
1	219	4	-19	3
**2**	115	1	-21	2
4	141	2	-43	2

Table S3: Determining the optimal concentration of βCD for the synthesis of cyclodextrin-modified selenium nanoparticles

mM CD	Diameter (nm)	SD	Zeta Potential	SD	%CD en NP by NMR
0	115	1	-21	2	0
1	153	7	-1	0	Not determined
8	195	2	-7.1	0.4	84

Table S4: **Determining the optimal concentration of the reducing agent, Sodium ~~(~~citrate (Ct) for the synthesis of gold nanoparticles**

mM Ct	Diameter (nm)	SD	Zeta Potential	SD	~~Se:V~~Au:Ct~~c~~ ratio
4	29	2	-26	2	1:2
**8**	**24**	**1**	**-13.2**	**0.4**	**1:4**
12	34	2	-4	1	1:6

Table S5: **Determining the optimal concentration of gold salt for the synthesis of gold nanoparticles**

mM $HAuCl_4$	Diameter (nm)	SD	Zeta Potential	SD
1	17.9	0.2	-7.4	0.9
**2**	**20.7**	**0.2**	**-6**	**2**
4	294	100	-19	2

Table S6: **Determining the optimal concentration of βCD for the synthesis of cyclodextrin-modified gold nanoparticles**

mM CD	Diameter (nm)	SD	Zeta Potential	SD	%CD en NP by NMR
0	20.7	0.2	-6	2	0
4	23.3	0.1	-23	2	Not determined
8	24.6	0.6	-20	2	73
12	19.9	0.1	-36	1	86
16	20.2	0.1	-37	1	Not determined
